# A simple and aesthetically pleasing floating craniotomy: How I do it

**DOI:** 10.1007/s00701-026-06827-1

**Published:** 2026-03-08

**Authors:** Tianzun Li, Qiumeng Li, Ji Xia, Yunpeng Dong, Mingliang Ren

**Affiliations:** Department of Neurosurgery, Daping Hospital, Army Medical University, Chongqing, China

**Keywords:** Decompressive craniectomy, Intracranial hypertension, Cranioplasty, Floating craniotomy, Hinged craniotomy

## Abstract

**Background:**

Decompressive craniectomy and subsequent cranioplasty are associated with significant morbidity (Kurland et al. Neurocrit Care 23:292–304, 2015). Bone flap-preserving techniques, including floating and hinged craniotomies, offer effective intracranial pressure (ICP) control (Mohan et al. Acta Neurochir (Wien) 163(5):1415–1422, 2021), although the optimal technique remains controversial, particularly in the context of traumatic brain injury where recent landmark trials have evaluated decompressive craniectomy outcomes (Patel et al. Trauma Surg Acute Care Open 10: e001784, 2025).

**Methods:**

The bone flap is loosely secured to the cranium using surgeon's knots and adjustable slip knots, with slip knot suture ends exteriorized through the scalp. Once elevated ICP resolves, the bone flap is definitively secured by tightening the externalized sutures.

**Conclusion:**

This straightforward floating craniotomy technique provides controlled decompression, effective ICP management, and preserved cosmesis.

**Supplementary Information:**

The online version contains supplementary material available at 10.1007/s00701-026-06827-1.

## Relevant surgical anatomy

The scalp comprises five distinct layers: skin, subcutaneous connective tissue, galea aponeurotica, loose areolar tissue, and pericranium. The loose areolar layer between the galea aponeurotica and pericranium provides an optimal plane for blunt dissection. Mobilization within this layer enhances scalp mobility and compliance.

The cranial vault consists of frontal, parietal, temporal, and occipital bones joined by sutures. Adult skull thickness averages 6–8 mm. In floating craniotomy, elevation of the bone flap expands intracranial volume [9], creating a buffer zone for mild to moderate brain swelling while maintaining bone protection and cosmetic appearance.

The Monro-Kellie doctrine dictates that the combined volume of intracranial blood, cerebrospinal fluid (CSF), and brain tissue must remain constant within the rigid cranial vault [6]. When pathological processes increase any component volume, compensatory mechanisms become exhausted, resulting in elevated ICP. A moderately elevated bone flap provides limited but clinically significant compensatory space.

## Description of the technique

Perform a standard large craniotomy with a bone flap measuring at least 12 × 15 cm [1]. Ensure adequate temporal decompression by removing the temporal squama and lesser sphenoid wing to the middle cranial fossa floor. Open the dura in stellate or curvilinear fashion. After intradural work is completed, if there is no significant brain swelling, or only mild swelling that does not exceed the cranial surface, and the ICP remains < 10 mmHg after replacing the bone flap and attempting to approximate the scalp [3], a floating bone flap procedure can be performed.

**Fig. 1:**
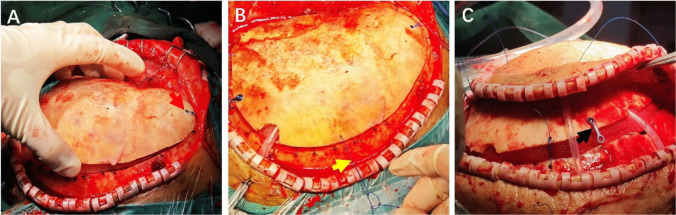
Intraoperative details of the bone flap fixation technique. **A** Frontal drill hole positioned approximately 1 cm from the bone flap edge, with suture forming a large loop after tying with a surgeon's knot (red arrow). **B** Slip knot with at least 2 cm of slack to allow outward bone flap excursion (yellow arrow). **C** One end of the titanium plate secured to the bone flap to prevent delayed flap subsidence (black arrow)

Using a 1-mm high-speed burr, create three small drill holes along the cranial edge with corresponding holes in the bone flap for suture passage. The three groups of drill holes are distributed approximately symmetrically on the skull, with one group in each of the frontal, parietal, and occipital regions. Place frontal holes approximately 1 cm from the bone flap and cranial edge. A nonabsorbable 0–0 Prolene suture is passed through the holes and tied with a surgeon's knot. The excess suture is then trimmed. This creates a relatively large loop allowing upward flap movement while preventing excessive displacement (Fig. 1A). At parietal and occipital positions, use slip knots with at least 2 cm of slack to permit outward bone flap excursion (Fig. 1B). Exteriorize both slip knot suture ends through the scalp.

Secure one end of a titanium plate to the bone flap with a screw at the parietal or occipital region to prevent delayed flap subsidence (Fig. 1C). Perform blunt subgaleal plane dissection around the incision to increase scalp mobility and compliance, creating additional space for outward flap movement.

**Fig. 2:**
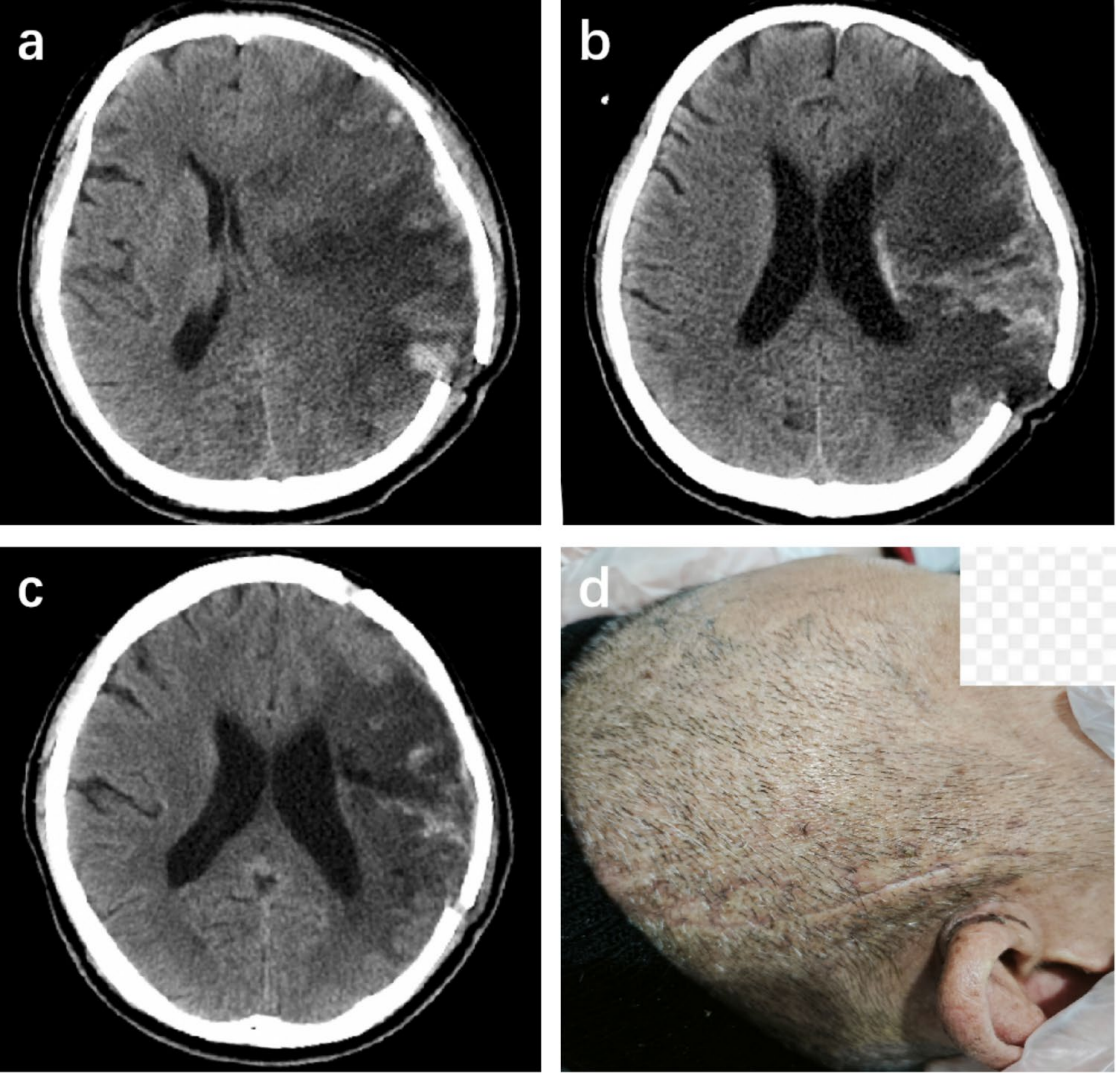
Postoperative imaging and clinical follow-up. **a** Head CT on postoperative day 3, demonstrating significant cerebral edema with good elevation of the floating bone flap. **b** Follow-up CT on postoperative day 9, showing resolution of cerebral edema while the bone flap remains elevated. **c** Follow-up head CT at 3 months, showing satisfactory anatomical reduction of the bone flap with no subsidence. **d** Clinical photograph at 3 weeks, showing an excellent cosmetic outcome with no signs of bone flap subsidence

Postoperatively, perform dressing changes every 2–3 days, maintaining wound cleanliness and dryness. According to clinical and imaging assessment (Fig. 2), once edema resolves, apply elastic compression bandaging to reposition the bone flap to its anatomical position, then tighten the slip knots to secure the flap to the surrounding cranium. To tighten a slip knot, stabilize one suture limb with a hemostat while using a second hemostat to gradually tension the contralateral limb. Continue to use the elastic compression bandage for 2–3 weeks after tightening the slip knot to ensure the bone flap is fully secured.

## Indications

This technique can be considered for patients who require surgical decompression but whose postoperative cerebral edema is not anticipated to be sufficiently severe to warrant a full decompressive craniectomy, such as in cases of traumatic intracranial hematoma [2].

## Limitations

Compared to standard decompressive craniectomy, this technique offers limited decompressive volume. It may prove inadequate for patients with fulminant or malignant cerebral edema, including those with large-area cerebral infarction [8].

## Complication avoidance

- Strictly select patients; avoid this technique in cases of severe brain swelling.
- Position drill holes away from major vessels in relatively avascular regions.
- Achieve meticulous hemostasis to prevent postoperative hemorrhage.
- Utilize protective postoperative positioning, avoiding pressure on the operative side.
- Continue ICP monitoring and ICP-directed management to detect abnormalities early.
- Prevent infection through strict aseptic technique, standardized antibiotic use, and diligent postoperative wound care.

## Specific perioperative considerations

Obtain comprehensive informed consent from the patient or legal guardian for incapacitated patients, including procedure details, objectives, and potential complications.

## Summary of key technical points

1. Create a bone flap with dimensions of at least 12 × 15 cm.
2. Remove the temporal squama and lesser sphenoid wing to the floor of the middle cranial fossa.
3. Incise the dura mater in a stellate or semicircular fashion and perform expansive duraplasty or cover with an artificial dural substitute.
4. Establish three suture fixation points for the floating bone flap, distributed symmetrically around the craniotomy defect.
5. Place frontal drill holes approximately 1 cm from the cranial bone edge.
6. Use monofilament nonabsorbable 0–0 Prolene suture exclusively for fixation.
7. Tie the frontal suture with a surgeon's knot to create a large loop, and use slip knots for the remaining sutures, allowing at least 2 cm of slack for flap elevation.
8. Tighten the slip knots promptly after edema resolution—usually within two weeks. Otherwise, consider cutting the exposed sutures and forgoing the knot.
9. Tighten slip knots by stabilizing one suture end while gradually applying tension to the sliding end.
10. Maintain sterile wound conditions postoperatively, paying special attention to the suture exit sites to prevent infection.

## Supplementary Information

Below is the link to the electronic supplementary material.

ESM 1 Supplementary Material 1 (MP4 477 MB)

## Data Availability

No datasets were generated or analysed during the current study.
